# HIV Testing and Treatment among HIV-Positive Men who have Sex with Men (MSM) Living in Russia: Data from Two Waves of the European MSM Internet Survey

**DOI:** 10.1007/s10461-024-04476-y

**Published:** 2024-09-02

**Authors:** Rigmor C. Berg, Vegard Skogen, Axel J. Schmidt, Roman Nesterov, Andrey Beloglazov

**Affiliations:** 1 UiT The Arctic University of Norway, Tromsø, Norway; 2 Norwegian Institute of Public Health, Oslo, Norway; 3 German AIDS Federation, Berlin, Germany; 4 London School of Hygiene and Tropical Medicine, London, UK; 5 Support for Social Initiatives and Public Health (SSIPH) Foundation, Moscow, Russian Federation

**Keywords:** MSM, HIV, ART, Russian Federation

## Abstract

We examined changes in HIV testing and medical care among men who have sex with men (MSM) in Russia. Data come from the 2010 and 2017 waves of the European MSM Internet Survey. From 2010 to 2017 there was an increase in the proportion who had ever received an HIV test (+ 11.2%), had tested for HIV in the last year (+ 2.1%), had ever taken antiretroviral therapy (ART) (+ 31.9), were currently taking ART (+ 31.5%), and had an undetectable viral load (+ 19.4%). These results are encouraging, yet they also reveal that substantial proportions of MSM experience considerable unmet prevention and treatment needs.

## Resumen

Examinamos los cambios en las pruebas de detección de VIH y la atención médica entre hombres que tienen sexo con hombres (HSH) en Rusia. Los datos provienen de las olas de 2010 y 2017 de la Encuesta Europea de Internet para HSH. De 2010 a 2017 hubo un aumento en la proporción de quienes alguna vez se habían hecho una prueba de VIH (+ 11.2%), se habían hecho una prueba de VIH en el último año (+ 2.1%), alguna vez habían tomado terapia antirretroviral (TAR) (+ 31.9%), estaban tomando TAR actualmente (+ 31.5%) y tenían una carga viral indetectable (+ 19.4%). Estos resultados son alentadores, pero también revelan que una proporción sustancial de HSH experimenta considerables necesidades de prevención y tratamiento no satisfechas.

## Introduction

More than 40 years into the epidemic, HIV is still a deadly pathogen, but for those having access to antiretroviral therapy (ART) it has become a chronic disease. Treatment is both lifesaving for people living with HIV and important in a public health perspective by preventing HIV transmissions. Yet, in the Russian Federation, the HIV epidemic has continued to grow for the past forty years and has become one of the world’s fastest growing HIV epidemics [1–3]. With about 1.1 million Russians out of 140 million living with HIV, Russia is estimated to have the largest number of people who are diagnosed with HIV of any country in Europe, accounting for over 80% of known infections in Eastern Europe and Central Asia [4, 5]. In 2022 alone, 55 573 Russians were diagnosed with HIV [5]. Although more than half of new HIV infections reported in the last few years resulted from unsafe heterosexual contact [5], the epidemic remains concentrated in key populations, including people who inject drugs (PWID), sex workers, and men who have sex with men (MSM) [3, 4]. Estimates suggest that about 15–20% of Russian MSM live with HIV [1]. Data on both prevalence and transmission routes are uncertain, however, and likely to underestimate sex between men as accounting for new diagnoses, because of legal and societal stigma against gay, bisexual, and other MSM, and laws banning even the sharing of information concerning homosexuality, including prevention information [2, 6].

Despite the high burden of HIV faced by MSM in Russia, their linkage to HIV care appears low. One of the few studies on the HIV continuum of care for MSM [6], with data collected in 2010, showed that among 1376 MSM in Moscow, 16% were HIV-positive but only 23% of these men reported being previously diagnosed and aware of their HIV-infection. Further, among those living with HIV, 17% reported being linked to care, 9% were currently on ART and 4% reported having an undetectable viral load [6]. These data are in stark contrast to recent Joint United Nations Programme on HIV/AIDS (UNAIDS) reported HIV continuum of care data for Russia overall. According to UNAIDS data, 72% of all people living with HIV in Russia knew their status, of these 78% were on ART, and of these, 75% had achieved viral suppression [4]. Similarly, a report from the European Centre for Disease Prevention and Control (ECDC) with 2022 data estimated that 69% of those with an HIV diagnosis had achieved viral suppression [5].

Because the UNAIDS and ECDC reported data do not disaggregate by mode of transmission and research among MSM in Russia is limited, it is not known whether HIV testing is reaching those at highest risk, such as MSM, and whether testing leads to knowledge of positive HIV status, access to medical care, and ultimately to viral suppression. We used the care continuum perspective [6] and aimed to examine the changes in self-reported HIV testing and medical care among MSM living with HIV in Russia, from 2010 to 2017. The data allow identification of impediments to engagement in care, initiation of ART, viral suppression and in turn possible strategies to address the expanding HIV epidemic among MSM in Russia.

## Methods

The data were collected as part of the European MSM Internet Survey (EMIS), an anonymous self-administered multi-language online survey that in 2010 and in 2017 recruited MSM from 38 to 50 countries, respectively, including Russia.

With EMIS-2017 being a replication of EMIS-2010 by the same research group, the two studies had similar objectives, methodology, and procedures. They were cross-sectional studies, with the objective to identify prevention needs commonly unmet across diverse groups of MSM. Promotion of the studies was through local, national, and international social-sexual networking apps and social media for gay, bisexual, and other MSM. The data were collected through an online, anonymous (neither names nor Internet Protocol addresses were collected), self-administered survey. Recruitment and data collection were from June to August 2010 and from October 2017 to January 2018. Eligibility criteria included being old enough to legally consent to have sex with men in the country of residence (age 16 in Russia), having male gender identity, being sexually attracted to men and/or having had sex with men and/or thinking he might have sex with men in the future, and providing informed consent. Participants received no recompense [7]. Detailed descriptions of the methods of EMIS-2010 and − 2017 are available on www.emis-project.eu.

In Russia, the national collaborating partners that effectuated recruitment were PSI Russia in 2010 and the charitable foundation Support for Social Initiatives and Public Health in 2017 – both non-governmental organizations in support of healthier lives for sexual minority populations. Along with EMIS-2010 and − 2017 study staff who commissioned advertising from multi-country online platforms, they promoted the surveys through advertisements on online dating sites and health organizations’ websites, and printed invitation cards and posters. In both waves, national promotion accounted for about 22% of respondents and the major online dating sites accounted for the rest, but while Qguys was the dating site that gave most returns in 2010 (34.1%), Hornet resulted in most returns in 2017 (48.7%). In both waves, almost all respondents answered the survey in Russian (98.1% in 2010 and 98.4% in 2017).

The survey was essentially identical in the two waves, based on several rounds of testing, and included tailored filtering (which depended on the respondent’s answer to previous questions; i.e., only respondents to whom the question applied received the question), such that fewer questions applied to each individual respondent. The final version of both surveys included mostly closed-ended questions, with answer options being largely recency scale, Likert scale, and binary (e.g., yes/no). The typical completion time was 20 min (auto-captured by the survey software).

To assess changes over time, of self-reported HIV testing and medical HIV care among MSM diagnosed with HIV, we obtained permission to use the data and selected the ten specific questions in both survey waves related to HIV and use of ART: whether the respondent had ever received an HIV diagnosis and recency of their last HIV test, the result of the most recent HIV test, the location of their initial HIV diagnosis (i.e., place, such as hospital), satisfaction with counselling when they were first diagnosed with HIV, recency of seeing a physician for monitoring their HIV infection, ever and current use of ART, viral suppression, and, if applicable (managed through tailored filtering), a multiple-choice question on reasons for never taking ART. Additionally, there were four general socio-demographics questions posed to all respondents, including age (continuous variable), education, employment status and sexual identity. We ran descriptive analyses and assessed the difference between the two samples of participants living in Russia (2010 *N* = 5035, 2017 *N* = 6247).

## Results

Table 1 presents the participants’ sociodemographic characteristics and HIV-related behaviors and treatment in the two waves of EMIS.

From 2010 to 2017 there was an increase in the proportion of MSM who had ever received an HIV test (74.3 vs. 85.5%, difference + 11.2), tested for HIV in the previous 12 months (66.9 vs. 69.0%, difference + 2.1), and there were more HIV-diagnosed respondents in the 2017 sample (13.4%) than the 2010 sample (3.6%). The two samples of HIV-diagnosed MSM were similar in sociodemographic characteristics, such as age.

Among MSM living with HIV, the proportion who had ever seen a physician for monitoring their HIV infection was rather similar in the two waves (“linked to care”; 94.2% and 95.4% in 2010 and 2017, respectively). Also the proportion who had seen a physician for monitoring their HIV infection in the previous six months was quite similar (“retained in care”; 86.1 vs. 89.4%, difference + 3.3). However, from 2010 to 2017 there was an increase in the proportion of MSM who lived with HIV who were currently taking ART (43.3 vs. 74.8%, difference + 31.5), and reported an undetectable viral load (34.2 vs. 53.6%, difference + 19.4). The main reasons for never having taken ART remained comparable in the two survey waves (more than one answer was possible): doctors had explained they do not need it yet (74.6 and 55.0%), respondent felt it was not necessary (20.3 and 10.5%), wanted to avoid side effects (12.1 and 11.1%), or did not want to be reminded (9.8 and 9.6%). In 2017, the answer option “I was diagnosed very recently” was added, and 33.9% of the respondents selected this answer option as their reason for never having taken ART.

**Table 1:** Description of the sample’s sociodemographic characteristics, HIV-related behaviors and treatment in EMIS-2010 and EMIS-2017

	EMIS-2010		EMIS-2017	
	*N*	%	*N*	%
**Full sample**	**5035**		**6247**	
Ever received an HIV test result	3741	74.3	5341	85.5
Tested for HIV in the previous 12 months	3368	66.9	4310	69.0
Diagnosed with HIV ever	316	3.6	834	13.4
Diagnosed with HIV, previous 12 months	47	0.9	212	3.4
**Sub-sample of MSM diagnosed with HIV**	**316**		**834**	
Age (median [mean and SD])	33 (33.5 [7.56])		33 (33.9 [8.37])	
Education				
High (ISCED 5–6) Mid (ISCED 3–4) Low (ISCED 1–2) (Missing)	219 86 10 (1)	69.5 27.3 3.2	503 250 44 (37)	63.1 31.4 5.5
Employment status				
Employed full- or part-time Unemployed Student Other (e.g. long-term sick leave, retired) (Missing)	275 15 7 18 (1)	87.3 4.8 2.2 5.7	714 53 32 33 (2)	85.8 6.4 3.8 4.0
Sexual identity				
Gay or homosexual Bisexual Straight or heterosexual Other/Don’t use a term	253 24 1 3	80.1 7.6 0.3 12.0	784 32 7 11	94.0 3.8 0.9 1.3
Place of initial HIV diagnosis				
Hospital or clinic as an out-patient General practitioner/family doctor At a community health service or drop-in (not in a hospital or clinic)^1^ A doctor in private practice At a hospital as an in-patient At a blood bank while donating blood I used a self-sampling/testing kit Elsewhere (Missing)	107 18 63 35 52 5 2 32 (2)	34.1 5.7 20.1 11.1 16.6 1.6 0.6 10.2	161 154 87 145 137 23 29 95 (3)	19.4 18.5 10.5 17.5 16.5 2.7 3.5 11.4
Satisfaction with counselling when first diagnosed				
Very satisfied/Satisfied Very dissatisfied/Dissatisfied None received Don’t remember/Unsure (Missing)	181 71 44 18 (2)	57.6 22.7 14.0 5.7	313 166 249 102 (4)	37.7 20.0 30.0 12.3
Continuum of HIV care				
Ever seen a physician for monitoring HIV (“linked to care”) (Missing)	291 (7)	94.2	795 (1)	95.4
Seen a physician for monitoring HIV, previous 6 months (“retained in care”) (Missing)	266 (7)	86.1	745 (1)	89.4
Ever taken ART (Missing)	141 (1)	44.7	611 (36)	76.6
Currently taking ART (Missing)	136 (2)	43.3	597 (36)	74.8
Have undetectable viral load	108	34.2	447	53.6
Reasons for never having taken ART:^2^	(175)		(223)	
I was diagnosed very recently	na		74	33.9
Doctor says I don’t need it yet	129	74.6	120	55.0
I feel it is not necessary	35	20.3	23	10.5
I want to avoid side effects	21	12.1	24	11.1
I don’t want to be reminded	17	9.8	21	9.6
I am afraid people will notice	6	3.5	15	6.9
Other reason	27	15.6	37	17.0
(Missing)	(2)		(5)	

Note: Number of missing answers provided for each variable when there were missing responses. Na = not an alternative that was provided in the survey. ISCED = International Standard of Classification Education (https://ec.europa.eu/eurostat/statistics-explained/index.php?title=International_Standard_Classification_of_Education_(ISCED)). 1 = the exact wording of this answer option was slight amended from “HIV testing service” (2010) to “community health service or drop-in” (2017). 2 = Eligible for the question were all who lived with HIV but never started or currently do not take ART (number in parenthesis)

## Discussion

We assessed the changes over time of self-reported HIV testing among MSM and the continuum of HIV care among MSM in the Russian Federation. Our study thus fills a gap and contributes to the limited literature on the alarming HIV situation among MSM in Russia [2], by finding that from 2010 to 2017, there was a positive trend in increased proportions of MSM testing for HIV, taking ART, and having an undetectable viral load.

From a care continuum perspective these results are encouraging. Russia appears to be making some progress in linking and retaining MSM in HIV care. Part of the reason may be the implementation of effective regular HIV testing across diverse settings, through both governmental institutions such as City AIDS Centers, and non-governmental organizations [8]. In 2022, 326 tests per 1000 population were performed [5]. Nonetheless, it should be noted that a considerable minority (26% and 14%) of our MSM respondents had never received an HIV test result. This indicates there may be challenges in accessing key populations within Russia for the purpose of conducting HIV testing. Noticeable also is that a considerable proportion of the MSM respondents in EMIS, in both waves, were very dissatisfied or dissatisfied with the counselling they received when they were first diagnosed with HIV. A bleak development documented in the results was that twice as many men in 2017, compared to 2010, received no counselling at all when they were first diagnosed. It has been previously noted that Russian HIV patients have restricted access to suitable HIV-related behavioral counselling [8] and qualitative studies reveal that some of the most commonly reported barriers of medical HIV care engagement are dissatisfaction with the services and negative attitudes of healthcare staff [9]. The lack of counselling and dissatisfaction with counselling may be part of the reason that a considerable portion of MSM diagnosed with HIV avoid or delay entering HIV care. We found that more than one in ten had not seen a physician for HIV monitoring in the previous six months. This indicates that about 10% of MSM diagnosed with HIV in Russia may be lost from care.

Perhaps most worrisome among the results was that in both study waves a considerable proportion had never taken ART, did not currently take ART and were not virally suppressed. A related rare study of Russian MSM living with HIV found that only 9% of MSM in Moscow were on ART in 2014 [6]. Although worrisome, the situation does not appear to be unique to MSM. A study among 227 Russian PWID who had lived with HIV for a mean of 7.3 years stated that only 19% had used ART before enrollment in the study. Moreover, during the 12-month study period, only 50.7% were linked to care [10]. Similarly, among a sample of HIV-positive men and women recruited in St. Petersburg between 2014 and 2018, about a quarter reported they were never offered ART. Among patients offered ART, one quarter declined or discontinued ART, and 45% were less than 95% adherent [11]. A recent analysis of national-level data obtained from four independent sources discovered that only 45% of all people diagnosed with HIV in Russia in 2018 received ART [3]. The EMIS-2010 and EMIS-2017 results indicate that Russian doctors may be reluctant to prescribe HIV-positive MSM with ART. More than half of the respondents stated that one of the reasons they had never taken ART was because their doctor had explained they did not need it yet. Similar reluctance to prescribe ART is reported in other studies [3, 9, 11], with one finding that 22% of people living with HIV had not been offered ART by their provider [11]. Reasons why a considerable proportion of MSM diagnosed with HIV are not successfully on ART regimens may in addition be attributable to Russia’s complex healthcare system, difficult bureaucracy governing access to counselling service and ART, stigma and lack of gay friendly treatment providers, and lack of trust in the quality of care and the public care system [1, 3, 11, 12].

The results must be interpreted in light of several limitations, including the cross-sectional nature of the data, which means the samples may not be fully comparable, and self-selection bias in the recruitment process as both samples are non-random samples that cannot be assumed to be representative of all MSM or all MSM living with HIV in Russia. Further, data are based on self-report. Limitations such as recall bias, social desirability bias, and measurement bias may affect the findings. Nonetheless, in conclusion, from the HIV care continuum perspective our study suggests that there are improvements in both HIV testing and medical care among MSM living with HIV in Russia. Yet, the results reveal that MSM experience considerable unmet prevention and treatment needs. To improve the situation for MSM, we advocate increasing access to HIV testing for MSM and referral of sexual partners, because it allows for both primary and secondary prevention [8]. Additionally, as others [2, 9, 11], we also support improved linkage of HIV-positive MSM to HIV care immediately upon diagnosis – which should include counselling – as well as making preexposure prophylaxis available and greater levels of ART coverage to maximize the effects of treatment as prevention. Reversal of the antigay propaganda law is similarly important to slow the spread of HIV among MSM, because such structural stigma influences HIV risk [1–3]. Given the rapid growth of the HIV epidemic, the ongoing COVID-19 pandemic, and the Russian–Ukraine conflict, we believe there is legitimate cause for concern that the positive trends among MSM may experience considerable setbacks.

## Data Availability

The dataset used and/or analysed during the current study are available from the corresponding author on reasonable request.

## References

[1] Amirkhanian Y. Emerging opportunities and challenges for HIV prevention, treatment, and care for MSM in the former Soviet Union and other post-communist states in Eastern Europe. Sex Transm Infect. 2017;93(5):305–6. 10.1136/sextrans-2016-052671. 28389443 10.1136/sextrans-2016-052671 PMC5522349

[2] Beyrer C, Wirtz AL, O’Hara G, et al. The expanding epidemic of HIV-1 in the Russian Federation. PLoS Med. 2017;14(11):e1002462. 10.1371/journal.pmed.1002462. 29182631 10.1371/journal.pmed.1002462 PMC5705067

[3] Nikoloski Z, King EJ, Mossialos E. HIV in the Russian Federation: mortality, prevalence, risk factors, and current understanding of sexual transmission. AIDS. 2023;37:637–45. 10.1097/QAD.0000000000003441. 36729857 10.1097/QAD.0000000000003441 PMC9994792

[4] UNAIDS, Country Factsheet. Russian Federation 2020 https://www.unaids.org/en/regionscountries/countries/russianfederation

[5] European Centre for Disease Prevention and Control/WHO Regional Office for Europe. HIV/AIDS surveillance in Europe 2023–2022 data. Stockholm: ECDC. 2023. https://www.ecdc.europa.eu/en/publications-data/hivaids-surveillance-europe-2023-2022-data

[6] Wirtz AL, Zelaya CE, Latkin C, et al. The HIV care continuum among men who have sex with men in Moscow, Russia: a cross-sectional study of infection awareness and engagement in care. Sex Transm Infect. 2016;92(2):161–7. 10.1136/sextrans-2015-052076. 26297721 10.1136/sextrans-2015-052076 PMC4889127

[7] Weatherburn P, Hickson F, Reid DS, et al. European men-who-have-sex-with-men internet survey (EMIS-2017): design and methods. Sex Res Soc Policy. 2020;17:543–57. 10.1007/s13178-019-00413-0.

[8] Lunze K, Cheng DM, Quinn EM, et al. Nondisclosure of HIV infection to sex partners and alcohol’s role: a Russian experience. AIDS Behav. 2013;17(1):390–8. 10.1007/s10461-012-0216-z. 22677972 10.1007/s10461-012-0216-z PMC3634358

[9] Kuznetsova AV, Meylakhs AY, Amirkhanian YA, et al. Barriers and facilitators of HIV care engagement: results of a qualitative study in St. Petersburg, Russia. AIDS Behav. 2016;20:2433–43. 10.1007/s10461-015-1282-9. 26767534 10.1007/s10461-015-1282-9 PMC4945482

[10] Dey AK, Ennis N, Cheng DM, et al. Impulsivity and linkage to HIV care among people living with HIV in St. Petersburg, Russia. AIDS Behav. 2022;26:4126–34. 10.1007/s10461-022-03738-x. 35708831 10.1007/s10461-022-03738-x PMC10202133

[11] Amirkhanian YA, Kelly JA, DiFranceisco WJ, et al. People living with HIV in St. Petersburg, Russia: gender and exposure group differences in HIV care engagement, psychosocial health, substance use, and transmission risk behavior. AIDS Educ Prev. 2022;34(3):226–44. 10.1521/aeap.2022.34.3.226. 35647864 10.1521/aeap.2022.34.3.226

[12] Stuikyte R, Barbosa I, Kazatchkine M. Getting to grips with the HIV epidemic in Russia. Curr Opin HIV AIDS. 2019;14(5):381–6. 10.1097/COH.0000000000000573. 31261159 10.1097/COH.0000000000000573

